# Championing the Dialysis Patient Voice: How has the US Legislation Fared?

**DOI:** 10.34067/KID.0000000000000560

**Published:** 2024-08-21

**Authors:** Osama El Shamy

**Affiliations:** Division of Renal Diseases and Hypertension, George Washington University, Washington, DC

**Keywords:** chronic dialysis, chronic hemodialysis, dialysis, health policy, hemodialysis, patient-centered care

There has been a growing emphasis on championing patient choices and priorities when managing medical conditions. The extent to which the patient voice is honored will always be dependent on the legislative restrictions by which medical professionals must abide, especially when it is directly tied to reimbursement.

Since the enactment of the Social Security Amendments of 1972, extending Medicare coverage to patients with ESKD, there has been a notable correlation between dialysis practice patterns in the United States and changes in legislative models. One can only assume that Congress did not expect ESKD-related costs to rise from $250 million in 1974 to $1 billion by 1979. Fast forward to 2021 and you will find that patients with ESKD comprise 1% of Medicare enrollment and 7% of its spending.^1^

There have been many policy changes and quality metric criteria that have been introduced along the way with the intention of supporting all patients with CKD, some of which have had unintended consequences and undermined patient autonomy. This perspective piece discusses some of these policies and explores their effects.

## High-Quality Care

In an attempt to incentivize high-quality care for patients on dialysis, the Centers for Medicare & Medicaid Services (CMS) changed the existing monthly capitation payment for physician reimbursement to a tiered fee-for-service model. As a result, physicians who saw their patients face-to-face up to four times a month would see an increase in their revenue. The intended consequence of this is for patients to receive higher quality care through incentivizing their medical providers to visit them more frequently. However, home dialysis payments remained capitated, resulting in lower potential reimbursements compared with their in-center counterparts.

### Unintended Result

While it did result in lower risk of incident hospitalization and reduced readmission rates, an analysis of patients on incident dialysis 3 years before and after the payment reform found that patients living in areas with large dialysis facilities saw a 16% reduction in the odds of being on home dialysis when compared with those living in areas with small facilities after the reform.^2^

## Prospective Payment System

With mounting fee-for-service costs—approximately 40% of the dialysis-associated costs^3^—Medicare introduced the prospective payment system (PPS) on January 1, 2011. Up until that point, facilities were reimbursed for their dialysis care regardless of modality, as well as a separate fee-for-service payment for drugs, laboratory testing, and supplies. Many small dialysis facilities relied on these fee-for-service charges to maintain revenues. The PPS was the first iteration of the affectionately coined “bundle” payment model. Dialysis providers would be reimbursed a single prospective payment, adjusting for patient and facility characteristics.

### Unintended Result

This incentivized dialysis providers to lower the overall cost of their therapies, motivating the use of lower cost drugs and dialysis modalities.^4^ Overall, there was a 0.5% increase in peritoneal dialysis utilization in 2011 after years of stagnation from 2007 to 2010.^3^ A first-year dialysis patient was 42% more likely to be on peritoneal dialysis in 2011 compared with 2007.^3^

Small chain-affiliated and independently owned dialysis facilities could no longer maintain revenues, and many merged with large dialysis organizations to benefit from their bulk discounts on medications, laboratories, and supplies, among other things.^5^ In fact, between 2006 and 2016, the percentage of small chain-affiliated and independently owned dialysis facilities decreased from 29% to 15%.^6^ A 2004 analysis^7^ found that large chain affiliation was an independent risk factor of patient mortality and that profit dialysis chains had a 13% higher risk of mortality.

Owing to a combination of minimizing costs, PPS, and Quality Incentive Program (QIP) (discussed later), erythropoietin and darbepoetin use declined 4.6% and the administration of cheaper iron formulations (*e.g*., iron sucrose) increased.^3^ There was also an overall decrease in vitamin D product use and increase in cinacalcet and phosphate binder usage. Average hemoglobin levels decreased and parathyroid hormone levels increased.

## Advancing American Kidney Health Initiative: Time to Get Patients Out of the In-Center Units

In 2019, the Advancing American Kidney Health (AAKH) initiative was signed with the ultimate goal of providing patients with kidney failure more options for treatment. One of the aims of the AAKH was that by 2025, 80% of patients with incident ESKD would either receive their dialysis at home or get a kidney transplant. This was shortly followed by the Home Dialysis Payment Adjustment and Performance Payment Adjustment, both introduced in January 2021. The former increased reimbursement rates for home dialysis, and the latter adjusted facility reimbursements on the basis of their home dialysis and transplant rates.

To support the aims of the AAKH, the End-Stage Renal Disease Treatment Choices model was introduced in 2021 whereby 30% of dialysis facilities were provided financial incentives for increasing the home dialysis usage and kidney transplantation.

### Unintended Result

The AAKH set an unrealistic target—a combined 16.2% of patients with incident ESKD^8^ in the United States were either started on home dialysis or had a preemptive kidney transplant in 2021. Furthermore, the payment adjustments will incentivize dialysis providers to expand their home dialysis capabilities, although a significant proportion of them are not certified to offer either home dialysis modality.^8^ While this may seem like a positive thing, an expansion of the desired scale is not possible with the current self-reported lack of competence in home dialysis management by graduating nephrologists and the severe nursing shortages seen across the dialysis industry, not to mention the scarcity of home dialysis–trained nursing workforce.^4^ Furthermore, an analysis of the first 2 years of the End-Stage Renal Disease Treatment Choices model found no significant increase in home dialysis usage or kidney transplantation in participating facilities compared with nonparticipating facilities.^9^

## End Stage Renal Disease QIP

To standardize the care of patients with ESKD across the United States, ESRD QIP was introduced in 2012 as the first federal value-based purchasing program. Given the varying landscape of dialysis providers—for-profit large dialysis organizations, not-for-profit organizations, and independent and hospital-based dialysis facilities—it is the ESRD QIP's goal to encourage dialysis facilities to provide high-quality care. Failure to meet the ESRD QIP measures could result in payment reductions of up to 2%. Therefore, meeting these metrics is imperative for dialysis providers to minimize their payment reductions.

The incorporation of the Consumer Assessment of Healthcare Providers and Systems in the QIP is important and provides patients the opportunity to evaluate nephrologists' communication and care, dialysis center staff, dialysis center care and operations, and the quality of information provided.

CMS’ determination that the Standardized Fistula Rate clinical measure does not provide patients and providers with the flexibility to choose the appropriate arteriovenous (AV) access was a step in the right direction. However, a facility's percentage of adult patients on hemodialysis using a catheter remains one of its quality measures. While there are exclusions, patient choice is not one of them.

Despite a dialysis provider's best efforts, there are patients who refuse to have AV access creation. Some have already had failed AV fistula creation or complications from the surgery, such as limb ischemia and steal syndrome, and are not willing to undergo another surgery. Rather than respecting the patient choice, some facilities weigh in the fact that this is a catheter patient when deciding whether to accept the patient into their facility.

A similar argument can be made regarding the CMS Kt/V requirement, given that small solute clearance alone is not a true measure of dialysis adequacy. Analysis of the CMS ESRD QIP data (payment year 2024) shows that only 52.9% of facilities achieved a Kt/V comprehensive rate of ≥90%.

## What Do Patients Want?

When asked to identify their priorities in hemodialysis, patients and caregivers identified fatigue/energy, resilience/coping, ability to travel, dialysis-free time, and effect on family as their top five priorities.^10^ Clinical parameters such as interdialytic weight gain, potassium, phosphate, calcium, parathyroid hormone and anemia were not even in the top ten priorities. This is not to minimize the effect of these metrics on patient outcomes, but what provisions do we have to incorporate patient priorities in our dialysis quality assessment guidelines?

It was previously shown that 45%–57% of patients who underwent predialysis education chose a home dialysis modality and approximately half of the patients who choose a home dialysis modality are started on their modality of choice. Only 3.1% of patients with incident ESKD received a preemptive kidney transplant in the United States in 2021.^8^ This puts the total percentage of incident ESKD patients who are willing to pursue home dialysis and/or getting a preemptive kidney transplant at a maximum of around 60%, far below the AAKH target of 80%. Rather than setting arbitrary targets, should our focus not be on increasing reimbursement for modality education services and making sure that those who want to go home end up going home? The AAKH sets a transplantation target. This is beyond the control of individual providers. Instead the focus should be on further incentivizing providers to list their patients for a kidney transplant.

Legislation is important. There will always be unintended consequences of executive decisions. It is a continuously evolving process and it is incumbent on nephrologists, nursing staff, dialysis providers, and—most importantly—patients having a say in what this legislation is advocating for, given that its intention is to provide patients with the highest quality care while lowering the ever-growing overall cost of caring for the ESKD patient population.
